# From Microbleeds to Iron: AI Prediction of Cerebrospinal Fluid Erythrocyte Load in Alzheimer’s Disease

**DOI:** 10.3390/jcm14207360

**Published:** 2025-10-17

**Authors:** Rafail C. Christodoulou, Georgios Vamvouras, Maria Daniela Sarquis, Vasileia Petrou, Platon S. Papageorgiou, Ludwing Rivera, Celimar Morales Gonzalez, Gipsany Rivera, Sokratis G. Papageorgiou, Evros Vassiliou

**Affiliations:** 1 Department of Radiology, Stanford University School of Medicine, Stanford, CA 94305, USA; 2 Department of Mechanical Engineering, National Technical University of Athens, 15772 Zografou, Greece; gvamvouras@mail.ntua.gr; 3 Department of Medicine, Universidad de Carabobo, Valencia 2001, Venezuela; mdsarquis58@gmail.com; 4 Department of Medicine, University of Ioannina, 45110 Ioannina, Greece; md07010@uoi.gr; 5 2nd Department of Orthopaedic Surgery and Traumatology, Aghia Sophia Pediatric General Hospital, Thivon 3 Street, 15772 Athens, Greece; pplaton24@gmail.com; 6 Department of Medicine, American University of Antigua, Jabberwock Road, Osbourn 999152, Antigua and Barbudacelimarm@auamed.net (C.M.G.);; 7 1st Department of Neurology, Medical School, National and Kapodistrian University of Athens, Eginition Hospital, 15772 Athens, Greece; 8 Department of Biological Sciences, Kean University, Union, NJ 07083, USA

**Keywords:** Alzheimer’s disease, artificial intelligence, red blood cells, explainable AI, amyloid, TAU

## Abstract

**Background/Objectives**: Cerebrospinal fluid erythrocyte load (CTRED) reflects occult red-blood-cell ingress into brain/CSF and consequent heme–iron exposure, a toxic pathway relevant to Alzheimer’s disease (AD). We aimed to develop explainable machine learning (ML) models that classify high vs. low CTRED from routine, largely non-invasive inputs, and to position a blood-first workflow leveraging contemporary plasma amyloid–tau biomarkers. **Methods**: Twenty-six ADNI participants were analyzed. Inputs were age, sex, mean arterial pressure (MAPres), amyloid (Aβ42), total tau, phosphorylated tau, and hippocampal atrophy rate (APC) derived from longitudinal MRI. APC was computed from normalized hippocampal volumes. CTRED was binarized at the median (0 vs. >0). Data were split into train (n = 20) and held-out test (n = 6). Five classifiers (linear SVM, ridge, logistic regression, random forests, and MLP) were trained in leakage-safe pipelines with stratified five-fold cross-validation. To provide a comprehensive assessment, we presented the contribution AUC, thresholded performance metrics, summarized model performance, and the permutation feature importance (PFI). **Results**: On the test set, SVM, ridge, logistic regression, and random forests achieved AUC = 1.00, while the MLP achieved AUC = 0.833. Across models, PFI consistently prioritized p-tau/tau, Aβ42, and MAPres; age, sex, and APC contributed secondarily. The attribution profile aligns with mechanisms linking BBB dysfunction and amyloid-related microvascular fragility with tissue vulnerability to heme–iron. **Conclusions**: In this proof-of-concept study, explainable ML predicted CTRED from routine variables with biologically coherent drivers. Although ADNI measurements were CSF-based and the sample was small, the framework is non-invasive by adding plasma p-tau217/Aβ1–42 for amyloid, tau inputs, and integrating demographics, hemodynamic context, and MRI. External, plasma-based validation in larger cohorts is warranted, alongside extension to MCI and multimodal correlation (QSM, DCE-MRI) to establish clinically actionable CTRED thresholds.

## 1. Introduction

Alzheimer’s disease (AD) is a progressive neurodegenerative disorder defined biologically by abnormal accumulation of β-amyloid (Aβ), tau, and clinically by insidious declines in memory, executive, and behavioral changes that can impair daily life [1]. It is the most common cause of dementia, affecting almost 7 million Americans aged 65 and older. Forecasts from the Global Burden of Disease (GBD) collaboration project claim a rise to 153 million by 2050, even as prevalence remains relatively stable [2]. A biomarker-based definition of AD was established by the National Institute on Aging and Alzheimer’s Association using the amyloid–tau–neurodegeneration framework, reflecting clinicopathological correlation and enabling earlier biologically grounded diagnosis [3,4].

Neuropathologically, AD features can be categorized into Aβ plaques and intracellular tau neurofibrillary tangles. This combination results in synaptic and neuronal loss, especially in vulnerable limbic temporal regions like the hippocampus and entorhinal cortex. Temporally, amyloid abnormalities typically emerge years to decades before symptoms (CSF Aβ42 decline and amyloid-PET positivity). Recent insights into the neuroimmune mechanisms of Alzheimer’s disease, along with earlier foundational work on the genetic and protein bases of amyloid pathology, reinforce this long preclinical phase and highlight opportunities for early intervention [5,6]. In contrast, tau pathology tracks clinical stage more closely and is a proximate driver of neurodegeneration [7]. Specifically, in multi-cohort tau-PET, uptake was able to explain over 40% of regional atrophy versus 3% in amyloid-PET. Therefore, the elevation of tau may locally precede neurodegeneration, further supporting its role in the downstream cascade of AD [8].

Extracellular β-amyloid deposition weakens cerebral vessel walls, producing cerebral amyloid angiopathy (CAA), and can also disrupt the BBB. Amyloid accumulates in leptomeningeal and cortical small vessels and predisposes to lobar microbleeds and superficial siderosis [9]. These microhemorrhages extravasate erythrocytes into the brain parenchyma and the cerebrospinal fluid (CSF). Their byproducts release heme and iron, which can start a process of oxidative stress and neuronal injury [10]. Prior studies have evaluated how RBC contamination affects CSF assays and cognition in AD, with varying lumbar puncture protocols and adjustments for RBC contamination (Table 1). Importantly, iron dysregulation is increasingly implicated in AD. Ferritin of CSF, a surrogate for brain iron-load, predicts cognitive decline and brain atrophy. Specifically, high vs. low ferritin levels were associated with a three-points-lower ADAS-cog 13 score, while high levels correlated with higher hippocampal atrophy (*p* = 0.02) [11]. Iron deposition and subsequent ferroptosis can influence the progression of various neurodegenerative diseases apart from AD. Multiple translational studies have shown that abnormal iron metabolism proteins in AD produce iron deposition and promote disease progression. Aβ and tau can also promote the deposition of iron in AD. Iron chelators’ effectiveness for treating AD further solidifies this mechanistic pathway [12].

The impaired blood–brain barrier (BBB) is an essential biomarker for early AD identification. The weakening of the BBB allows for greater passage of molecules and damage in the cerebral microvasculature, which leads to microbleeds [13]. Tau pathology can impair the BBB by tau-driven inflammation and pericyte/endothelial injury. High mean arterial pressure (MAPres) can be associated with microbleeds in the context of small vessel disease, but it can also enhance the disruption of the BBB. DCE-MRI shows BBB impairment in early AD [14]. CSF markers of barrier injury, such as PDFFRβ (pericyte injury), tau/p-tau, and ANGPT-2, reflect endothelial activation and barrier dysfunction [15].

Demographic features further influence these relationships. Age increases the burden of small vessel disease and the prevalence of microbleeds, while also raising the likelihood of barrier breakdown by disrupting tight proteins, making RBC leakage more common in older patients than younger ones [16,17]. Tauopathy and amyloidopathy between sexes have clear divergence and can be further modified by the status of menopause. Buckley (2022) et al. showed in the Framingham PET study that post-menopausal women have higher Tau (*p* < 0.002) and Aβ (*p* = 0.010) PET uptake compared to middle-aged men [18]. These data support the idea that modeling age and sex can influence the molecular pathophysiology of AD and result in higher levels of red blood cells in CSF (CTRED).

AD diagnostics are shifting toward minimally invasive blood-based biomarkers. In May 2025, the U.S. FDA cleared the first plasma assay to aid in diagnosing AD pathology (Lumipulse G p-tau217/Aβ1–42 plasma ratio), reflecting strong concordance with amyloid-PET/CSF [19]. In the meantime, studies have shown that plasma p-tau217 can match CSF biomarkers for identifying biological AD across clinical settings (accuracy up to 91%), including primary care, enabling scalable screening and longitudinal monitoring [20]. Representative applications of ML to AD cohorts are summarized in Table 2.

Building on this framework, machine learning (ML) data-driven algorithms that learn patterns from examples have been increasingly used to predict AD biomarkers and clinical outcomes from routinely acquired, non-invasive inputs. Because clinical adoption requires transparency, explainable AI (XAI) approaches are favored so clinicians can see which variables drive predictions [21]. Therefore, in our model, we employed permutation feature importance (PFI), which showed MAPres, tau, and Aβ42 as the most significant predictors, followed by age, sex, and hippocampal atrophy, which contributed to a lesser degree, aligning with the current AD literature.

Notably, prior iron-focused work did not use ML; it reported statistical associations showing that CSF ferritin, a proxy for brain iron, relates to Aβ/tau pathways and predicts cognition and atrophy [11]. That literature establishes biological plausibility for an iron axis in AD. Still, it leaves the question of whether we can infer iron exposure, specifically red-blood-cell-derived heme–iron from microbleeds, using accessible clinical data.

Our ML models predict CTRED with an AUC starting at 0.833, indicating occult RBC leakage and iron exposure, using minimally invasive clinical, imaging, and blood data, including FDA-cleared plasma biomarkers. By modeling contributors such as BBB permeability, amyloid burden (Aβ42), tau (p-tau), demographics, and APC targets, a complex clinical outcome is typically achieved, often requiring lumbar puncture (LP). Though ADNI traditionally measured Aβ/tau via LP, current plasma assays allow the same model to run non-invasively with blood, vitals, and MRI, predicting CTRED. This creates a new link from microbleeds to iron, adding a vascular–iron dimension to amyloid/tau/neurodegeneration staging, while remaining interpretable and deployable.

**Table 1:** Studies of RBC load and their impact on AD patients: cognition or biomarkers.

Study (Year)	Cohort (Size and Characteristics)	CSF Collection and Processing	RBC Measurement	Key Findings (RBC vs. Cognition or Biomarkers)	Reference
**Ayton et al. (2015)**	ADNI (302: 91 CN, 144 MCI, 67 AD; 7-y follow-up)	LP at ADNI sites standardized freezing	CSF Hb as proxy for RBC load	Elevated CSF ferritin predicted worse cognition and faster MCI → AD conversion; findings adjusted for CSF Hb to avoid RBC bias.	[11]
**Ayton et al. (2018)**	ADNI (296 with CSF ferritin; annual biomarker follow-up 5 y)	Standard ADNI LP	CSF Hb	High CSF ferritin (>6.2 ng/mL) predicted accelerated CSF Aβ42 decline in amyloid-positive subjects; no tau association.	[22]
**Konen et al. (2023)**	Patients without AD (N = 29)	Atraumatic LP; discard 1st 2 mL; no centrifugation; analysis ≤30 min; spiking with 10,000–20,000 RBC/µL ± storage 14 d at 4 °C	Autologous blood spiking verified by counter	Core AD biomarkers (Aβ42, t-tau, p-tau) stable even with 20,000 RBC.	[23]
**Summary** Direct RBC-focused AD studies are limited, and handling methods vary between research protocols. AD biomarkers robust to RBC contamination with modern assays. Gaps: standardized protocols, plasma-based validation, accounting for vascular/tap confounds.					

**Table 2:** Machine learning approaches in AD.

Study (Year)	Data (Cohort and Modality)	ML Model and Features	Performance	Interpretability	Reference
**El-Sappagh et al. (2021)**	ADNI (N = 1,048,294 cognitively normal, 486 MCI, 268 AD multimodal: demographics, APOE, CSF biomarkers, MRI, PET)	Two-layer random forest (diagnosis + MCI conversion)	Accuracy 93.9% (dx), 87.1% (MCI conversion); AUC 0.90	SHAP + 22 surrogate explainers (decision trees, fuzzy rules)	[24]
**Yue et al. (2023)**	CLAS (N = 2.658 elderly; lifestyle + clinical data)	Ensemble ML (9 classifiers + 5 feature-selection methods)	AD vs. HC: 99.2%; MCI vs. HC: 89.2%	SHAP global + patient-level explanations	[25]
**Grammenos et al. (2024)**	ADNI MCI subset (240 patients) plasma biomarkers + MRI hippocampal atrophy	XGBoost + feature selection	85% accuracy cross-validation	SHAP dependence plots features: p-tau, Aβ42, hippocampal volume	[26]
**Jiao et al. (2025)**	Multicenter Chinese cohorts (N = 1324); plasma digital biomarkers design from ATR-FTIR features)	Random forest	AD vs. HC AUC 0.92; AD vs. DLB AUC 0.83; AD vs. FTD AUC 0.80	Spectral peaks identified; correlated with p-tau217 and GFAP	[27]
**Summary** ML widely applied to AD biomarkers and diagnosis with high performance. RBC/iron biomarkers (CTRED) largely absent. Gaps: small cohorts, generalizability, regulatory validation.					

## 2. Materials and Methods

Data were obtained from the ADNI dataset (http://adni.loni.usc.edu, accessed on 3 September 2025). The ADNI project was launched in 2003 as a public–private partnership with the primary goal of testing whether clinical, imaging, genetic, and biochemical biomarkers can be combined to measure the progression of mild cognitive impairment (MCI) and early Alzheimer’s Disease (AD). All participants gave written informed consent for data collection and sharing during enrolment. The study protocols and consent forms were approved by each participating institution’s institutional review boards (IRBs). Descriptive statistics concerning the utilized portion of the dataset can be seen in Table 3:

In this study, five machine learning (ML) models were used to classify CTRED levels as high or low, using as input the annual percentage change (APC, defined in Equation (2)) in normalized averaged hippocampal volume (Equation (1)), age, sex, MAPres (Equation (3)), p-tau, tau, and Aβ42.

(1) $$hipp_{norm}=\frac{Left\;Hippocampus+Right\;Hippocampus}{2*total\;brain\;volume}$$

(2) $$APC=\frac{last\;hipp_{norm}\;measurement-first\;hipp_{norm}\;measurement}{first\;hipp_{norm}\;measurement}*\frac{100\%}{years\;between\;scans}$$

(3) $$MAPres=\frac{3\cdot DBP+2\cdot SBP}{3}$$

**Figure 1:**
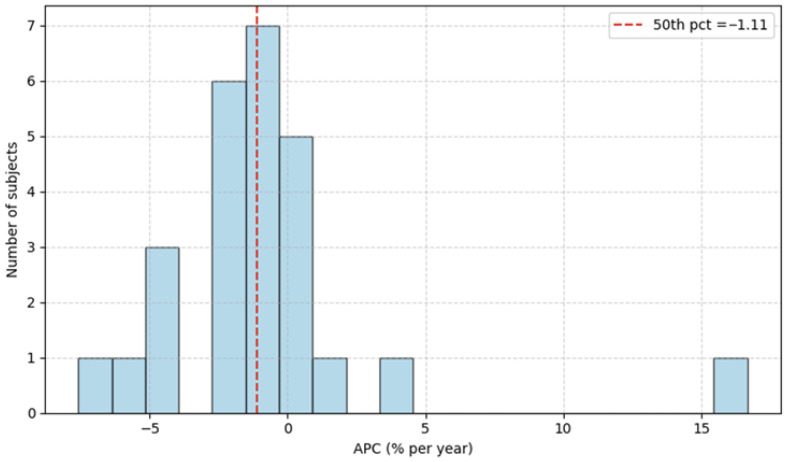
Distribution of APC values.

The left and right hippocampus volumes and the total brain volume were measured from MRI modalities using FreeSurfer, as described in [28]. Τwenty-six subjects were available. The final APC variable was converted to binary, labeled as low- and high-risk, based on the original APC value. Specifically, subjects above the 50th percentile were labeled as high-risk, whereas those below the 50th percentile were labeled as low-risk, as seen in Figure 1. The corresponding APC value at the 50th percentile is −1.11%, and low-risk subjects are denoted by label 0, contrary to higher-risk subjects, denoted by label 1. The data is split into a training set (20 subjects) and a testing set (6 subjects).

Additionally, CTRED values were converted to binary, labeled as low and high, based on the median of CTRED = 0, meaning that approximately half the subjects had CTRED = 0 and the other half had CTRED > 0. Although this separation appears simplistic, it suffices to capture the underlying trends given the small sample size of merely 20 train and 6 test samples.

### Models and Hyperparameter Optimization

Five model architectures were tested using Python v.3.12.3 (tensorflow v.2.19.0, scikit-learn v.1.4.2, optuna v.4.4.0, numpy v. 1.26.4, keras v.3.10.0). The first four, linear SVM, ridge classifier, logistic regression classifier, and random forests classifier, were linear, whereas the fifth was a small MLP.

1. SVM Model description

The SVM model used a linear kernel, and all features were standardized within a pipeline to avoid data leakage. The classifier is an SVC (kernel = “linear”, probability = True, class_weight = “balanced”). A stratified 5-fold CV (shuffled, fixed seed) was used for training. In each fold, the full pipeline (scaler + SVM) fits on the fold’s training split and is evaluated on its held-out split. Fold-level predictions were aggregated to compute AUC from decision scores (margins) and standard classification metrics from labels. Because probability = True, probabilities are obtained via Platt scaling applied to the SVM’s decision scores within scikit-learn.

2. Hyperparameter optimization

The linear SVM’s regularization C was optimized using Optuna [29], sampling C from the log-uniform range [10^−3^, 10^3^]. For each trial, we trained a leakage-safe pipeline (StandardScaler → SVC with linear kernel, probability = True, class_weight = “balanced”) and selected the model by stratified 5-fold cross-validated F1-score, which balances precision/recall under class imbalance. After identifying the best C, the pipeline was refit on the whole training set, while the parameters and the final model were saved for inference. Margins (decision scores) were used for AUC computation.

3. Ridge Classifier Model description

A RidgeClassifier (L2-regularized linear classifier) was employed within a leakage-safe pipeline (StandardScaler then RidgeClassifier, class_weight = “balanced”). Inputs were standardized inside the pipeline. Training and evaluation were conducted with stratified 5-fold cross-validation (shuffled, with fixed seed). Because RidgeClassifier does not expose calibrated probabilities, ranking performance was assessed via the decision function for AUC. After cross-validation, the model was refit on the whole training set, and AUC was computed from decision scores of the final fitted model rather than fold-averaged probabilities.

4. Hyperparameter optimization

The L2 regularization strength α was optimized with Optuna over a log-uniform range [10^−3^, 10^3^]. A leakage-safe pipeline (StandardScaler then RidgeClassifier, class_weight = “balanced”) was trained and evaluated with stratified 5-fold CV for each trial. The objective maximized the negative squared error cost, defined in Equation (3), computed from each fold’s confusion matrix, thereby directly penalizing false positives and false negatives quadratically. After the optimal α was identified, the model was refit on the full training set for reporting. Because RidgeClassifier does not expose calibrated probabilities, AUC was computed from decision scores rather than predict_proba.

5. Logistic Regression Classifier Model description

Logistic regression with L2 regularization was applied inside a pipeline (StandardScaler then LogisticRegression, class_weight = “balanced”). Training and evaluation used a stratified k-fold CV with shuffling and a fixed seed. Because logistic regression outputs calibrated probabilities through the sigmoid, AUC was computed directly from predicted probabilities. Labels were assigned using a tuned threshold rather than the default 0.5, by sweeping out-of-fold predictions on the training set.

6. Hyperparameter optimization

Optuna tuned solver, penalty type, regularization strength C, maximum iterations, and class weighting. The search spanned solvers {liblinear, saga}, penalties {L1, L2}, *C* ∈ [10^−3^, 10^3^] (log-uniform), and max iterations 500–5000. Each trial trained a leakage-safe pipeline and was evaluated by a stratified cross-validated ROC AUC. The best parameters were retrained on the full training set, and the final threshold was determined separately from the training set’s out-of-fold probabilities.

7. Random Forests Classifier Model description

Random forest classifiers were built inside a pipeline with class_weight = “balanced_subsample”. Training used stratified k-fold CV with shuffling and fixed seed. Outputs were taken as class probabilities, and thresholds were tuned by sweeping cutoffs under constraints on the positive prediction rate to avoid degenerate solutions. AUC was computed directly from predicted probabilities, while labels were based on the optimized threshold.

8. Hyperparameter optimization

Optuna optimized forest size, depth, split, leaf constraints, feature sampling, bootstrap use, and criterion. Search ranges included 100–800 estimators, depths 2–12, min split 2–10, min leaf 1–6, and feature selection {sqrt, log2, all}. Each trial produced out-of-fold probabilities, and the objective selected thresholds to maximize a custom score that penalized false positives and negatives. The final model was based on the full training set with the best parameters.

9. MLP Model description

An MLPClassifier (feed-forward neural network) was employed within a leakage-safe pipeline (StandardScaler then MLPClassifier). Inputs were standardized inside the pipeline. Training and evaluation were conducted with stratified 5-fold cross-validation (shuffled, fixed seed). For each fold, the full pipeline was fit on the training split and evaluated on the held-out split. Fold-level probabilities were aggregated to compute AUC, and labels were used for standard classification metrics and confusion matrices. Because MLPClassifier exposes calibrated class probabilities via predict_proba, ranking performance was assessed directly from those probabilities. After cross-validation, the model was refit on the whole training set.

10. Hyperparameter optimization

Hyperparameters were optimized with Optuna. The search space included the following: n_layers in {1, 2}, n_hidden_1 i [4, 32] (log scale), n_hidden_2 in [4, 32] (log scale, used only if n_layers = 2), activation in {relu, tanh, logistic, solver in {adam, sgd}, alpha in *α* ∈ [10^−6^, 10^−1^] (log scale), learning_rate_init ∈ [10^−4^, 10^−1^] (log scale), batch_size = “auto”, and early_stopping enabled. Each trial trained a leakage-safe pipeline (StandardScaler then MLPClassifier) and was scored by stratified 5-fold CV. The objective maximized the mean of the score defined in Equation (3) across folds, directly penalizing false positives and false negatives quadratically. After the best configuration was identified, the pipeline was refit on the full training set and both the parameter dict and the final pipeline were saved for inference.

## 3. Results

### 3.1. SVM Model

#### 3.1.1. Classification Results

**Figure 2:**
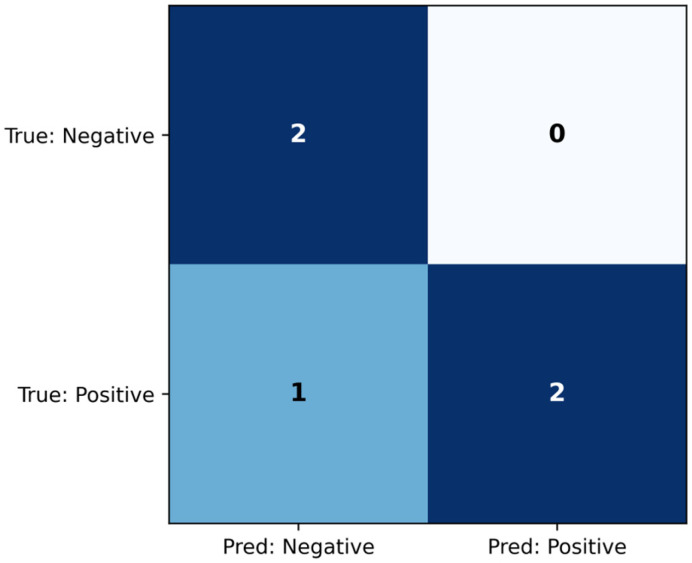
Confusion matrix for the SVM model, evaluated on the test set.

The linear SVM model was evaluated on the test set comprising six samples. The confusion matrix is seen in Figure 2, and the classification metrics are shown in Table 4. The model achieved AUC = 1.000, which indicates that it distinguished between the classes perfectly. Additionally, the classification metrics demonstrate that the threshold chosen on the training set also led to successful classification on the held-out test set.

#### 3.1.2. Explainability

**Figure 3:**
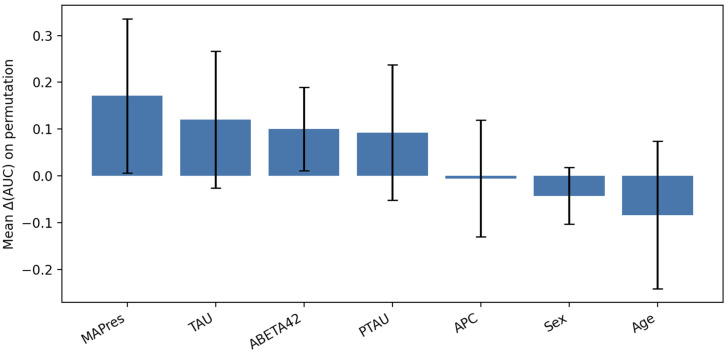
PFI for SVM classifier, evaluated on the train set.

Permutation feature importance (PFI) was used to quantify predictive contribution by repeatedly permuting each feature within stratified 5-fold cross-validation and recording the mean change in AUC with standard deviations. This analysis (Figure 3) indicated that MAPres and Tau significantly affected model discrimination. At the same time, Aβ42 and p-tau contributed moderately, and APC, Sex, and Age had a negligible or unstable impact.

### 3.2. Ridge Classifier

#### 3.2.1. Classification Results

**Figure 4:**
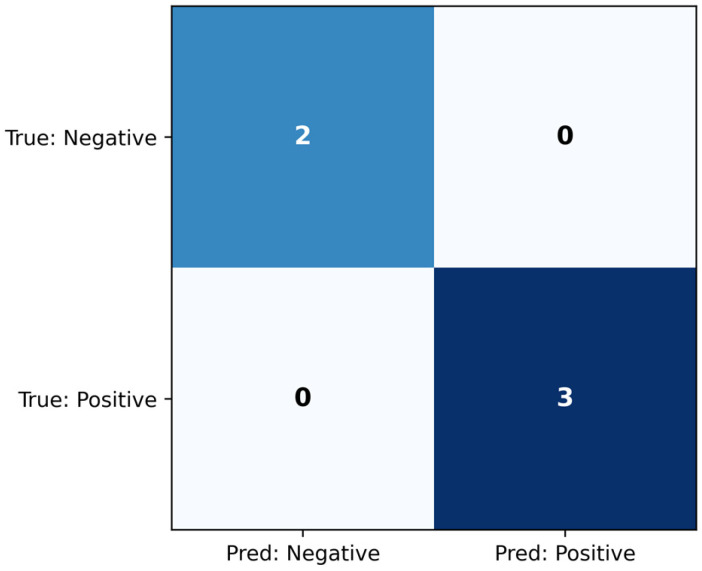
Confusion matrix for the ridge classifier, evaluated on the test set.

The ridge classifier was evaluated on the test set, and the confusion matrix is seen in Figure 4, while the classification metrics are shown in Table 5. The model achieved AUC = 1.000, which indicates that it managed to distinguish between the classes perfectly. Additionally, the classification metrics demonstrate that the threshold chosen on the training set also led to successful classification on the held-out test set.

#### 3.2.2. Explainability

**Figure 5:**
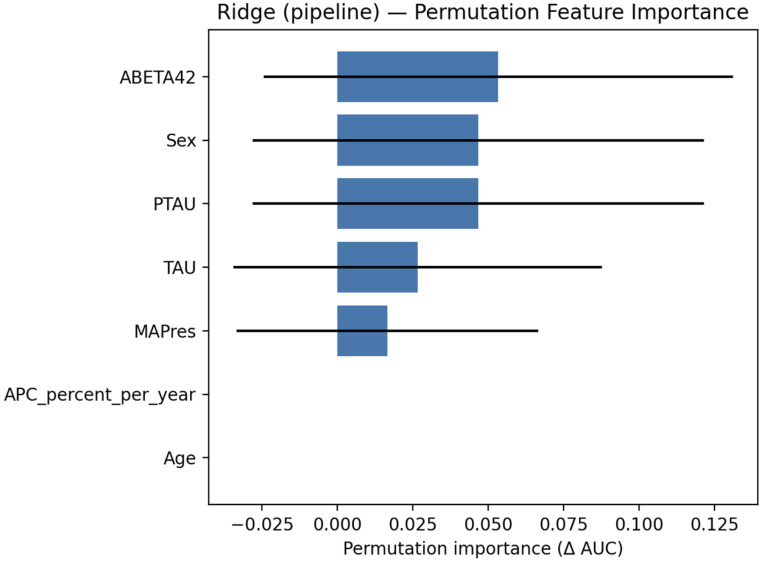
PFI for the ridge classifier, evaluated on the train set.

Permutation feature importance (PFI) was used by permuting features within stratified 5-fold cross-validation and recording the mean change in AUC with variability estimates. The most substantial contributions were observed for Aβ42, sex, and p-tau, while tau and MAPres showed more minor effects, and APC, together with patients’ age, had negligible importance (Figure 5).

### 3.3. Logistic Regression Classifier

#### 3.3.1. Classification Results

**Figure 6:**
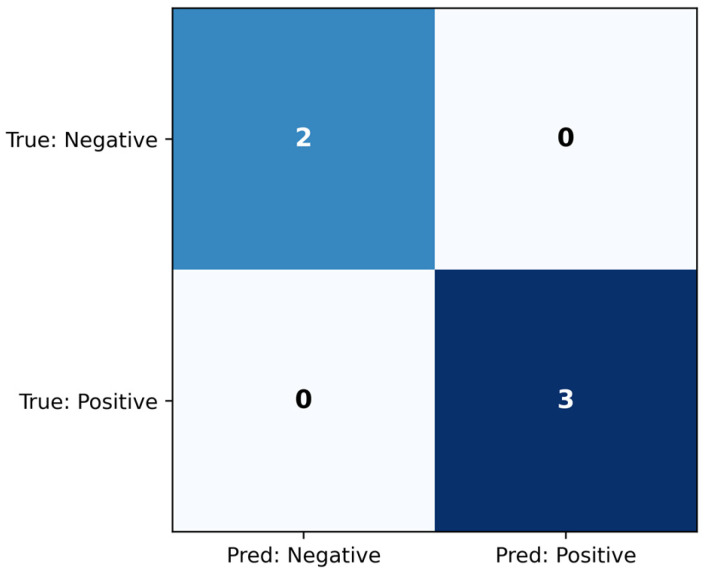
Confusion matrix for the logistic regression classifier, evaluated on the test set.

The logistic regression classifier was evaluated on the test set. The confusion matrix is seen in Figure 6, while the classification metrics are shown in Table 6. The model achieved AUC = 1.000, which indicates that it distinguished between the classes perfectly. Additionally, the classification metrics demonstrate that the threshold chosen on the training set also led to successful classification on the held-out test set.

#### 3.3.2. Explainability

**Figure 7:**
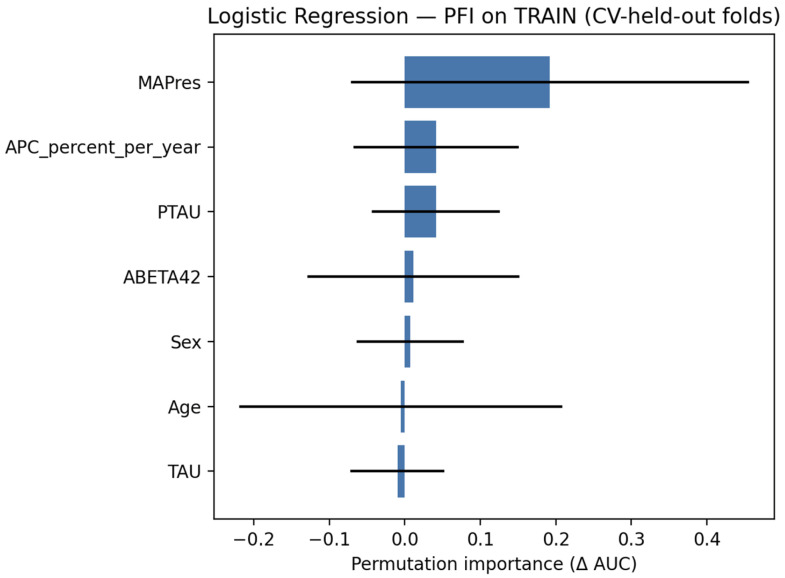
PFI for the logistic regression classifier, evaluated on the train set.

Permutation feature importance (PFI), as seen in Figure 7, indicated that MAPres contributed most strongly, with more minor effects from p-tau and APC, while the remaining features were of limited relevance.

### 3.4. Random Forests Classifier

#### 3.4.1. Classification Results

**Figure 8:**
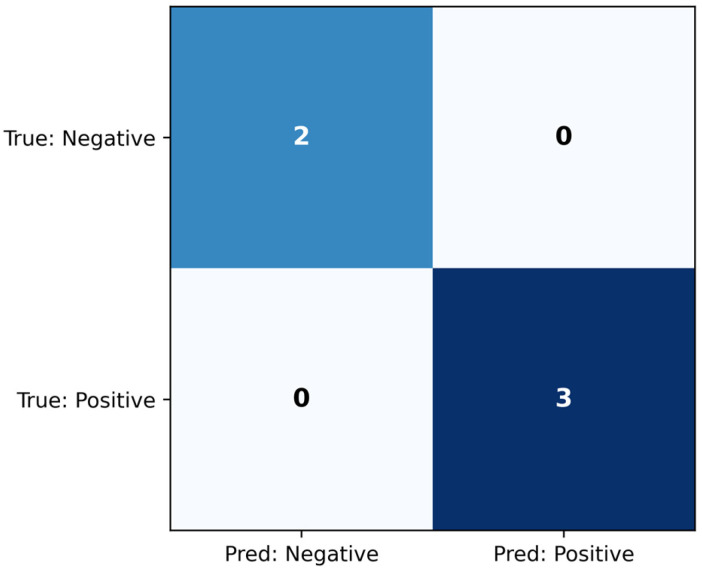
Confusion matrix for the random forests classifier, evaluated on the test set.

The random forests classifier was evaluated on the test set, and the confusion matrix is shown in Figure 8 while the classification metrics are shown in Table 7. The model achieved AUC = 1.000, which indicates that the model managed to distinguish between the classes perfectly. Additionally, the classification metrics demonstrate that the threshold chosen on the training set also led to successful classification on the held-out test set.

#### 3.4.2. Explainability

**Figure 9:**
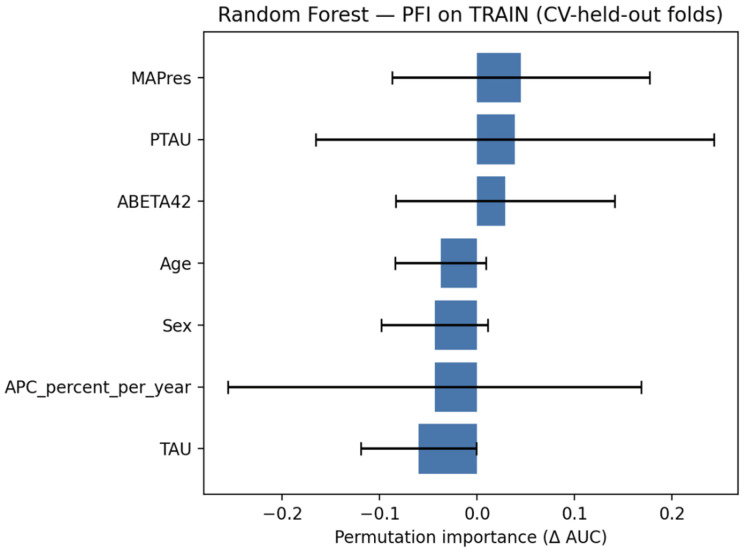
PFI for the random forests classifier, evaluated on the train set.

Permutation feature importance (PFI), seen in Figure 9, was estimated in stratified cross-validation, showing MAPres and p-tau as the most influential variables, with Aβ42 also contributing, and age, sex, APC, and tau exerting weaker or unstable effects.

### 3.5. MLP Classifier

#### 3.5.1. Classification Results

**Figure 10:**
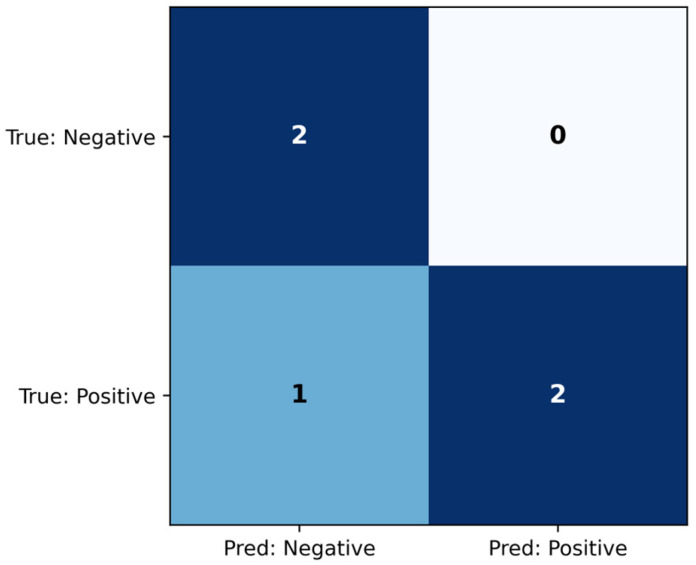
Confusion matrix for the MLP classifier, evaluated on the test set.

The MLP classifier was evaluated on the test set, and the confusion matrix is shown (Figure 10), while the classification metrics are shown in Table 8. The model achieved AUC = 0.833, which indicates that the model distinguishes the two classes well. Additionally, the classification metrics demonstrate that all true negatives were identified from the six test samples, and only one true positive was misclassified as negative.

#### 3.5.2. Explainability

**Figure 11:**
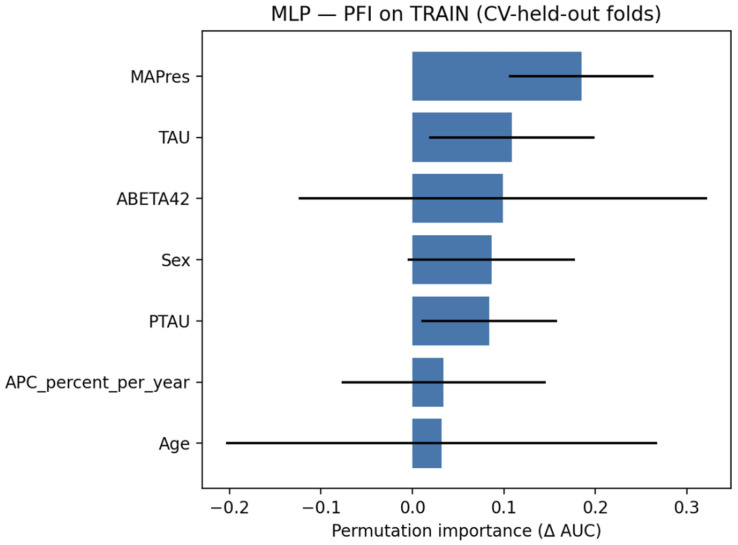
PFI for the MLP classifier, evaluated on the train set.

Figure 11 shows that permutation feature importance (PFI) indicated that MAPres was the most influential variable, with tau and Aβ42 following. Sex and p-tau contributed moderately, and APC, together with age, showed minimal effects.

## 4. Discussion

### 4.1. Principal Findings

Five supervised ML classifiers were used to predict CTRED as high or low based on routine inputs (MAPres, age, sex, Aβ42, t-tau, p-tau, hippocampal APC). On an independent test set (n = 6), four models achieved perfect discrimination (AUC = 1.00) using linear SVM, ridge, logistic regression, and random forests, while the MLP obtained an AUC of 0.833. Confusion matrices and thresholded metrics showed similar results; SVM and the other models yielded perfect precision and recall on the six test cases. At the same time, the MLP misclassified one positive (precision 1.00, recall 0.67, F1 = 0.80). Due to the minimal test set, these metrics are optimistic and should be interpreted cautiously. However, cross-validated training performance and consistent feature-importance patterns suggest a genuine link between vascular, amyloid, and tau biology with RBC leakage into CSF.

### 4.2. Mechanistic Interpretability and Biological Relevance

**Figure 12:**
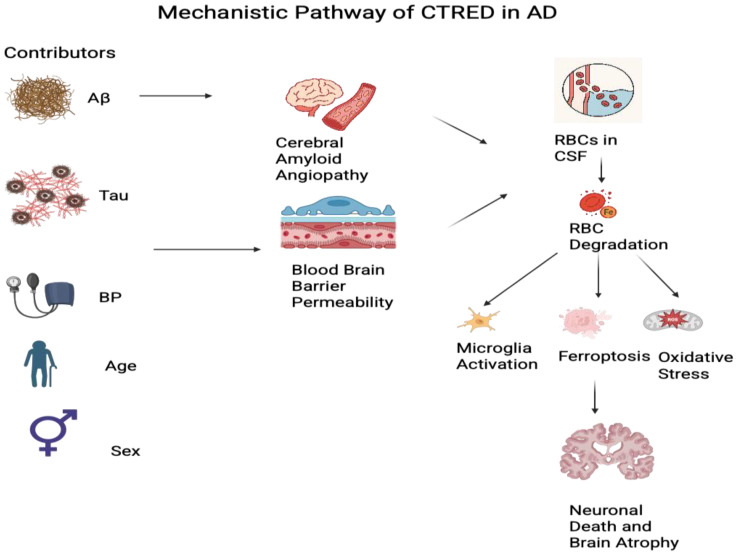
Mechanistic pathway of CTRED in Alzheimer’s disease.

Explainability analyses converged on a coherent mechanistic picture in which driver factors of CTRED reflect two complementary roles for RBCs’ ingression into the CSF (Figure 12). The first can be explained by hemodynamic stress and BBB failure. In contrast, the second can be explained by amyloid-related angiopathy vessel fragility, where Aβ predisposes to microbleeds and superficial siderosis. Age and sex have been shown to modulate these pathways through various mechanisms. At the same time, APC reflected a downstream vulnerability in a region that AD had affected earlier. High MAPres can cause vessel-wall remodeling, making it thicker and more rigid [30]. When combined with chronic strain, this can lead to wall disruption and microbleeds. Population-based studies have linked elevated BP to a gradual accumulation of red blood cells within CSF over time.

Additionally, elevated levels of MAPres can cause BBB impairment through various mechanisms. Damage to small vessels is linked to BBB dysfunction. Moreover, increased strain on endothelial cells increases their permeability. The connection between hypertension and oxidative stress can further accelerate BBB impairment [31]. Notably, Kerkhof (2024) et al. [32] demonstrated that 22 patients with hypertension exhibited a BBB on 7T DCE-MRI before detectable brain parenchyma damage. Effective treatment was associated with reduced BBB disruption, indicating that antihypertensive medications may offer protective effects [32].

The prominence of tau (t-tau/p-tau) in feature rankings is biologically plausible for two reasons. First, BBB disruption correlates with tau-related pathology and pericyte/endothelial injury in AD patients’ CSF studies. Markers of pericyte damage, such as sPDGFRβ and ANGPT-2, remain elevated in tau pathology, leading to a barrier-injury environment that promotes RBC leakage. Specifically, a positive statistically significant correlation in sPDGFRβ was observed with total tau (*p* = 0.007) and phosphorylated tau (*p* = 0.013) [14,15]. Second, tau burden aligns more closely with local neurodegeneration and cognition across different cohorts than amyloid. Tau-PET has shown higher SUVR uptakes in multiple cognitive domains, whereas amyloid showed few or non-specific associations. Mediation analyses show that neurodegeneration partly explains the tau–cognition link, with tau also impacting independently of atrophy and Aβ, indicating its role as a direct dysfunction driver. While Aβ influences the extent of the tau–cognition relationship, the regional tau signal is the primary clinical correlate [33,34]. This clarifies why our models consider weighted p-tau/tau together with Aβ, as tau indicates regions of active neurodegeneration and is associated with BBB/pericyte damage. At the same time, amyloid contributes to microvascular weakness. This intersection likely results in higher CTRED, which our classifiers are specifically trained to predict.

Amyloid-related processes in AD are highly complex. CAA may be the key explanation for the initial origin of CTRED. CAA involves the deposition of Aβ40 (type 2) and Aβ42 (type 1). The predominance of Aβ42 in type I, known as capillary CAA in leptomeningeal and cortical capillaries, weakens these vessels, increasing the risk of bleeding. This process can lead to lobar microbleeds and superficial bleeding siderosis [9,35]. Recent studies have shown the link of Aβ42, a surrogate for amyloid plaque burden, to be responsible for BBB disruption. Experimental evidence shows Aβ injures neurovascular cells and damages BBB integrity [36]. In vitro and animal studies corroborate human results when brain microvessels are exposed to Aβ, which leads to damage and junction disruption by triggering ROS in pericytes and speeding up their demise [37]. Therefore, this highlights the significant role of amyloid burden in our models, as it can alter CTRED levels through two distinct and fundamental mechanisms.

Once erythrocytes breach the brain or CSF and lyse, hemoglobin is broken down into heme and ferrous iron (Fe^2+^), increasing the labile iron pool [38]. This redox activity promotes the formation of reactive oxygen species, lipid peroxidation, and microglial activation and can lead to ferroptosis [12,39]. These hemoglobin/heme pathways are documented in human neurological disease and justify measuring CSF CTRED as a proxy for heme–iron exposure. CSF ferritin, an indicator of brain iron-stores, predicts cognitive decline and hippocampal atrophy and can be explained by Aβ42 and tau, linking iron biology to the amyloid/tau framework. Notably, CSF ferritin levels were predicted using regression analysis by Aβ1–42 (*p* = 0.029) and tau levels (*p* < 0.001). These findings are consistent with prior studies that examined how cerebral microbleeds or iron load interact with AD biomarkers, as summarized in Table 9. Our approach extends this to infer RBC-derived iron exposure (CTRED) using explainable ML from routine, minimally invasive inputs.

Age and sex may act as modifiable factors. Increased age can weaken the BBB due to endothelium degeneration, lower microvascular density, and decreased pericytes. Then, it raises the occurrence of microbleeds, heightening the chance of RBC ingress and higher CTRED [40]. Sex differences, such as greater tau burden in amyloid-positive women and vascular changes related to menopause, may also influence risk [8,41]. Higher tau accumulation in menopausal women could be attributed to low estrogen levels, since they can prevent the cleavage of tau proteins and reduce the formation of neurofibrillary tangles [42]. These findings align with the more minor yet plausible impacts of age and sex seen in our models.

Moreover, hippocampal APC, an MRI-based measure of long-term atrophy, probably indicates tissue vulnerability downstream [43]. The hippocampus can be susceptible to BBB disruption due to its high metabolic demand, making it vulnerable to biological insults (iron toxicity) [44]. As a secondary factor in our models, its trend aligns with areas where BBB failure and heme–iron exposure (high CTRED) lead to observable neurodegeneration.

Contributors, including Aβ, tau, BP, age, and sex, target vascular and barrier-related processes like cerebral amyloid angiopathy and BBB permeability. These events facilitate RBC entry into the cerebrospinal fluid (CSF), leading to RBC breakdown and release of heme and iron. The effects include microglial activation, ferroptosis, and oxidative stress, which ultimately cause neuronal death and brain atrophy.

**Table 9:** Studies of cerebral microbleeds or iron load concerning AD biomarkers.

Study (Year)	Sample/Cohort	Iron/CMB Measurement	AD Biomarker(s)	Key Findings	Reference
**Christodoulou et al. (2025)**	ADNI; AD group n = 18 (25 observations), CN group included	CSF erythrocyte burden (CTRED); MRI entorhinal volume	CSF p-tau181	CTRED predicted a smaller entorhinal volume (*p* = 0.005). Significant p-tau × CTRED interaction (*p* = 0.004): higher CTRED amplified p-tau-related atrophy.	[45]
**Oomens et al. (2025)**	Multi-cohort ABS; N = 4080 (CN, MCI, AD)	MRI CMBs (lobar vs. deep)	Amyloid status (PET or CSF Aβ)	Amyloid-positive individuals had more lobar CMBs. Risk increased further with APOE ε4 and age.	[46]
**Rauchmann et al. (2020)**	ADNI; N = 189 (CN, MCI, AD)	MRI microhemorrhage count (T2 *-GRE)	Amyloid-PET, Tau-PET, CSF Aβ42/t-tau/p-tau	A higher microbleed burden correlated with higher Aβ-and tau-PET at baseline and predicted longitudinal increases in Aβ (global/parietal) and tau (parietal).	[47]
**Romero et al. (2020)**	Framingham Heart Study	MRI CMB presence (lobar vs. deep, GRE)	Plasma Aβ40/42, plasma tau	Higher plasma Aβ40 is associated with lobar CMBs (CAA-related). Higher plasma tau associated with any CMB. No effect for Aβ42/40.	[48]
**McCarter et al. (2022)**	Mayo Clinic Study of Aging; N = 712 adults	MRI CMB count (GRE)	Plasma Aβ42/40, t-tau, p-tau181, p-tau217; Amyloid-PET (subset)	More CMBs linked to lower plasma Aβ42/40. In those with ≥2 CMBs, higher plasma p-tau217 strongly associated with higher amyloid-PET uptake.	[49]
**van Bergen et al. (2016)**	Zurich aging study: N = 37 (22CN and 15 MCI), APOE genotyped	7T MRI QSM (cortical iron)	Amyloid-PET (11C-PiB)	Cortical iron strongly co-localized with amyloid plaques; effect more pronounced in APOE ε4 carriers.	[50]
**Ayton et al. (2018)**	ADNI; N = 296 (CN, MCI, AD)	CSF ferritin	CSF Aβ42, tau (longitudinal)	High ferritin predicted faster decline in CSF Aβ42 (faster plaque accumulation). No association with tau.	[22]
**Ayton et al. (2023)**	BioFINDER + ADNI replication; N = 1239	CSF ferritin	CSF p-tau181, A/T classification; cognition	Higher ferritin associated with elevated p-tau181 (APOE-mediated). Ferritin cutoff predicted faster cognitive decline in MCI.	[51]
**Spotorno et al. (2020)**	BioFINDER-2; N = 236 Aβ-positive (prodromal AD + AD dementia)	3T MRI QSM (cortical iron)	Tau-PET (18F-RO948);	Regional iron correlated with tau-PET signal. Mediation showed iron partly explained tau’s effect on cortical atrophy’s stronger effects in younger patients.	[52]
**Summary** **Consistent pattern:** Higher CMB burden or iron load associates with higher Aβ and/or tau pathology. APOE ε4 often amplifies these effects. **Gaps:** Evidence is largely observational; causal pathways and stage-specific effects remain to be clarified.					

### 4.3. Explainability and Face Validity

PFI within stratified cross-validation measures each variable’s contribution by the drop in model discrimination when that feature is permuted. Across different models, PFI consistently highlights p-tau/tau and amyloid (using Aβ42 as the amyloid marker) along with MAPres, with age, sex, and APC playing secondary roles.

This attribution profile appears valid as higher weights are assigned to variables related to BBB dysfunction and amyloid-associated microvascular fragility. At the same time, tau indicates tissue vulnerability, especially where heme–iron exposure is critical, matching the biology. At the patient level, the model can provide a brief ranked list of key drivers (e.g., “p-tau ↑, Aβ42 ↓, MAPres ↑”), allowing clinicians to review why a “high CTRED” label was assigned and assess its consistency with the clinical picture. The alignment of these explanations with known mechanisms enhances clinician trust and facilitates practical integration of the tool into reporting workflows.

### 4.4. Clinical Adaptability and Translational Value

**Figure 13:**
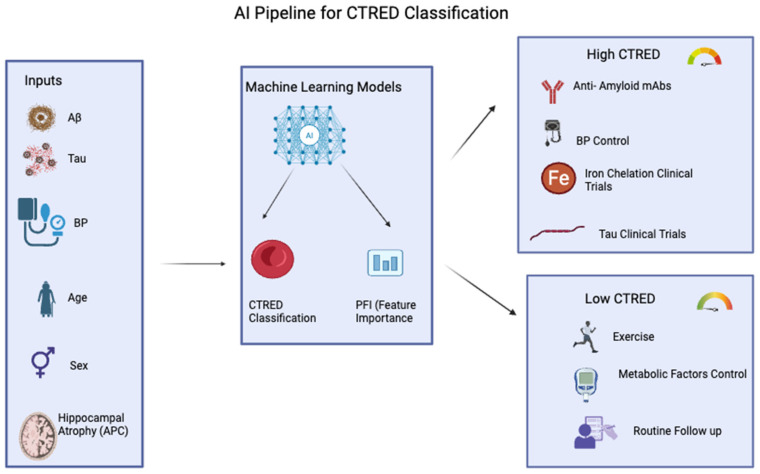
AI pipeline for CTRED classification.

The non-invasive pipeline requires routine demographic data (age, sex), MRI-derived hippocampal atrophy rate (APC), and blood-based amyloid/tau biomarkers. Notably, the FDA-approved plasma p-tau217/Aβ1–42 ratio allows confirmation of amyloid and tau levels without lumbar puncture (LP) or PET scans, enabling this model to adopt a “blood-first” approach, where feature values directly map 1:1 from plasma assays to the model’s inputs. This allows us to measure CTRED non-invasively instead of using invasive methods like LP (Figure 13) [53]. Notably, predictions accompanied by PFI enable clinicians to identify which variables contributed to a “high CTRED” designation and explain the reasoning during patient care. In practice, a high CTRED label with amyloid and tau as primary indicators facilitates discussions about anti-amyloid monoclonal antibodies (such as lecanemab and donanemab) in accordance with the Appropriate Use Recommendations for early AD, which include biomarker confirmation and ARIA risk management [54,55]. When hemodynamic load contributes (high MAPres), the PFI report justifies tighter blood-pressure control given established links between hypertension and cerebral microbleeds. Because age and sex are non-modifiable, they inform counseling rather than intervention. If CTRED remains high after addressing modifiable drivers, clinicians may consider research referral for iron-modulating strategies; current randomized data with deferiprone lowered hippocampal iron in QSM but worsened cognition, so chelation should remain trial-only [56].

Conversely, a low-CTRED result supports routine risk-factor management (lipid profile, diabetes, BP, activity) and ongoing follow-up [57]. Given AD’s multifactorial nature, CTRED predictions and driver readouts are meant to guide multidisciplinary decisions rather than replace clinical judgment.

The pipeline integrates demographic data (age, sex), MRI-derived hippocampal atrophy rate (APC), and plasma biomarkers (Aβ, tau, and p-tau217/Aβ1–42 ratio) as model inputs. The machine learning model outputs CTRED classification (high vs. low) with permutation feature importance (PFI) highlighting the most influential predictors. High CTRED results guide therapeutic considerations, including anti-amyloid monoclonal antibodies, blood pressure control, and potential enrollment in iron chelation or tau-targeted clinical trials. Low CTRED results support non-pharmacologic strategies such as exercise, metabolic risk-factor management, and routine follow-up.

### 4.5. Limitations

The study has some limitations. First, the small cohort (n = 26; train 20/test 6) likely yields optimistic, uncertain point estimates, especially the perfect test AUCs. The CTRED target was median-binarized, simplifying continuous biology. Despite all quality precautions taken during the ADNI protocol study to prevent contamination during LP, some blood contamination could not be avoided or excluded. The results should be considered preliminary evidence due to the limited sample size and the need for further validation with larger, independent datasets, including calibration and decision-curve analysis, to improve generalizability beyond this proof-of-concept.

Furthermore, the model depends on costly, sensitive longitudinal MRI volumetry, which may be less accessible and vary by scanner/protocol, limiting reproducibility in some clinical settings. Although ADNI labels were CSF-based, our deployment aims for blood-first plasma p-tau/Aβ, requiring prospective plasma validation. Finally, while we evaluated both simple linear models and slightly more complex multilayer perceptrons (MLPs), the strong performance of interpretable linear models suggests that additional algorithmic complexity may not be necessary, and future work should prioritize approaches that balance accuracy with scalability and transparency. Ultimately, clinical translation of such models will depend not only on the model’s accuracy but also on the accessibility of plasma biomarkers, reproducibility across scanners and populations, explainability to build clinician trust, and alignment with regulatory approval pathways.

### 4.6. Future Directions

Future research will broaden CTRED profiling across the MCI spectrum to better understand its timing and dose–response relationship. This involves modeling continuous CTRED (not just high/low) against hippocampal APC, cognition, and incident dementia within a larger longitudinal cohort, and validating plasma-based markers (p-tau217/Aβ1–42) across different scanners and sites to ensure non-invasive applicability.

Simultaneously, we will link CTRED to brain iron and BBB function by correlating it with QSM (tissue iron measurement) and DCE-MRI Ktrans (permeability). We aim to establish a clinical CTRED threshold where patients exhibit higher QSM and faster APC, then explore eligibility for iron-chelation trials as an initial step. To understand mechanisms better, Ktrans and QSM will be stratified by three upstream factors: amyloid burden (Aβ42 or Aβ42/40, PET when available), tau load (plasma p-tau, tau-PET for validation), and hemodynamic load (MAPres) to identify phenotypes where barrier failure, vascular-amyloid fragility, and tau-linked vulnerability intersect with elevated CTRED. These directions will guide BP management, anti-amyloid strategies, and research into iron-targeted interventions.

## 5. Conclusions

This proof-of-concept study developed explainable machine learning models to predict CTRED, a marker of occult red-blood-cell leakage and iron exposure in Alzheimer’s disease, using non-invasive inputs. Across five models, high classification performance was observed with PFI rankings highlighting biologically plausible contributors like p-tau/tau, amyloid burden (Aβ42), and MAPres. At the same time, demographic and atrophy measures provided secondary signals. This profile aligns with mechanisms linking vascular fragility, tau pathology, and iron neurotoxicity.

Clinically, a blood-first workflow using the FDA-approved plasma p-tau217/Aβ1–42 ratio, demographics, MRI APC, and hemodynamic data positions CTRED prediction as a non-invasive, explainable tool. It could help stratify patients by iron-exposure risk, aiding decisions on vascular, anti-amyloid, tau, or iron-modulating interventions. Despite limitations from small sample size and reliance on MRI volumetrics, our framework paves the way to develop CTRED models for MCI, incorporate QSM and DCE-MRI data, and define thresholds for iron-related neurotoxicity.

Overall, predicting that CTRED links amyloid–tau neurodegeneration to a vascular–iron dimension creates a scalable, interpretable bridge “from microbleeds to iron” in Alzheimer’s disease biomarkers and therapies.

## Figures and Tables

**Table 3:** Cohort Characteristics.

Characteristic	Values
Age mean (SD)	73.8 (7.6)
Sex	
Male	11
Female	15
APC yearly % change (mean, SD)	−0.9, (4.3)
CTRED (mean, SD)	121.5 (526.8)
MAPres (mean, SD)	92.3 (8.7)

**Table 4:** SVM model classification metrics, evaluated on the test set.

	Precision	Recall	F1-Score
**Class 0**	0.667	1.000	0.800
**Class 1**	1.000	0.667	0.800

**Table 5:** Ridge classification metrics, evaluated on the test set.

	Precision	Recall	F1-Score
**Class 0**	1.000	1.000	1.000
**Class 1**	1.000	1.000	1.000

**Table 6:** Logistic regression classification metrics, evaluated on the test set.

	Precision	Recall	F1-Score
**Class 0**	1.000	1.000	1.000
**Class 1**	1.000	1.000	1.000

**Table 7:** Random forests classification metrics, evaluated on the test set.

	Precision	Recall	F1-Score
**Class 0**	1.000	1.000	1.000
**Class 1**	1.000	1.000	1.000

**Table 8:** MLP classification metrics, evaluated on the test set.

	Precision	Recall	F1-Score
**Class 0**	0.667	1.000	0.800
**Class 1**	1.000	0.667	0.800

## Data Availability

The data presented in this study are available on request from the Alzheimer’s Disease Neuroimaging Initiative (ADNI) database: http://adni.loni.usc.edu, accessed on 3 September 2025. Access to the data requires registration and compliance with ADNI’s data-use agreement. The authors did not generate any new datasets in this study.

## Abbreviations

The following abbreviations are used in this manuscript:

AD	Alzheimer’s Disease
ADAS-cog	Alzheimer’s Disease Assessment Scale—Cognitive Subscale
ADNI	Alzheimer’s Disease Neuroimaging Initiative
AI	Artificial Intelligence
APC	Annual Percentage Change
Aβ	Amyloid-beta
Aβ42	Amyloid-beta 1–42
ANGPT-2	Angiopoietin-2
AUC	Area Under the Curve
BBB	Blood–Brain Barrier
BP	Blood Pressure
CAA	Cerebral Amyloid Angiopathy
CSF	Cerebrospinal Fluid
CTRED	Cerebrospinal Fluid Total Red Blood Cell Erythrocyte Load
CV	Cross-Validation
DCE-MRI	Dynamic Contrast-Enhanced Magnetic Resonance Imaging
FDA	Food and Drug Administration
F1	F1-score (harmonic mean of precision and recall)
HR	Heart Rate
IRB	Institutional Review Board
L2	L2 Regularization (ridge/logistic regression)
MAB(s)	Monoclonal Antibody(/ies)
MAPres	Mean Arterial Pressure (residual, modeled feature)
MCI	Mild Cognitive Impairment
ML	Machine Learning
MLP	Multilayer Perceptron
MRI	Magnetic Resonance Imaging
PET	Positron Emission Tomography
PFI	Permutation Feature Importance
QSM	Quantitative Susceptibility Mapping
ROC	Receiver Operating Characteristic
ROS	Reactive Oxygen Species
sPDGFRβ	Soluble Platelet-Derived Growth Factor Receptor Beta
SUVR	Standardized Uptake Value Ratio
SVM	Support Vector Machine
t-tau	Total Tau
p-tau	Phosphorylated Tau
XAI	Explainable Artificial Intelligence

## References

[1] Alzheimer’s Association 2025 Alzheimer’s Disease Facts and Figures. https://www.alz.org/getmedia/ef8f48f9-ad36-48ea-87f9-b74034635c1e/alzheimers-facts-and-figures.pdf.

[2] Nichols E., Steinmetz J.D., Vollset S.E., Fukutaki K., Chalek J., Abd-Allah F., Abdoli A., Abualhasan A., Abu-Gharbieh E., Akram T.T. (2022). Estimation of the global prevalence of dementia in 2019 and forecasted prevalence in 2050: An analysis for the Global Burden of Disease Study 2019. Lancet Public Health.

[3] Braak H., Braak E. (1991). Neuropathological stageing of Alzheimer-related changes. Acta Neuropathol..

[4] DeTure M.A., Dickson D.W. (2019). The neuropathological diagnosis of Alzheimer’s disease. Mol. Neurodegener..

[5] Jorfi M., Maaser-Hecker A., Tanzi R.E. (2023). The neuroimmune axis of Alzheimer’s disease. Genome Med..

[6] Selkoe D.J. (2001). Alzheimer’s disease: Genes, proteins, and therapy. Physiol Rev..

[7] Bateman R.J., Xiong C., Benzinger T.L., Fagan A.M., Goate A., Fox N.C., Marcus D.S., Cairns N.J., Xie X., Blazey T.M. (2012). Clinical and Biomarker Changes in Dominantly Inherited Alzheimer’s Disease. N. Engl. J. Med..

[8] Malpetti M., Joie R.L., Rabinovici G.D. (2022). Tau Beats Amyloid in Predicting Brain Atrophy in Alzheimer Disease: Implications for Prognosis and Clinical Trials. J. Nucl. Med..

[9] Greenberg S.M., Van Veluw S.J. (2024). Cerebral Amyloid Angiopathy. Stroke.

[10] Zhang M., Cheng Y., Zhai Y., Yuan Y., Hu H., Meng X., Fan X., Sun H., Li S. (2022). Attenuated iron stress and oxidative stress may participate in anti-seizure and neuroprotective roles of xenon in pentylenetetrazole-induced epileptogenesis. Front. Cell. Neurosci..

[11] Ayton S., Faux N.G., Bush A.I., Alzheimer’s Disease Neuroimaging Initiative (2015). Ferritin levels in the cerebrospinal fluid predict Alzheimer’s disease outcomes and are regulated by APOE. Nat. Commun..

[12] Wang F., Wang J., Shen Y., Li H., Rausch W.-D., Huang X. (2022). Iron Dyshomeostasis and Ferroptosis: A New Alzheimer’s Disease Hypothesis?. Front. Aging Neurosci..

[13] Wang H., Zhang C., Qiu Y., Chen A., Li Y., Hu B. (2021). Dysfunction of the Blood-brain Barrier in Cerebral Microbleeds: From Bedside to Bench. Aging Dis..

[14] Van De Haar H.J., Burgmans S., Jansen J.F.A., Van Osch M.J.P., Van Buchem M.A., Muller M., Hofman P.A., Verhey F.R., Backes W.H. (2016). Blood-Brain Barrier Leakage in Patients with Early Alzheimer Disease. Radiology.

[15] Miners J.S., Kehoe P.G., Love S., Zetterberg H., Blennow K. (2019). CSF evidence of pericyte damage in Alzheimer’s disease is associated with markers of blood-brain barrier dysfunction and disease pathology. Alzheimer’s Res. Ther..

[16] Poels M.M.F., Vernooij M.W., Ikram M.A., Hofman A., Krestin G.P., van der Lugt A., Breteler M.M. (2010). Prevalence and Risk Factors of Cerebral Microbleeds: An Update of the Rotterdam Scan Study. Stroke.

[17] Andjelkovic A.V., Situ M., Citalan-Madrid A.F., Stamatovic S.M., Xiang J., Keep R.F. (2023). Blood-Brain Barrier Dysfunction in Normal Aging and Neurodegeneration: Mechanisms, Impact, and Treatments. Stroke.

[18] Buckley R.F., O’Donnell A., McGrath E.R., Jacobs H.I.L., Lois C., Satizabal C.L., Ghosh S., Rubinstein Z.B., Murabito J.M., Sperling R.A. (2022). Menopause Status Moderates Sex Differences in Tau Burden: A Framingham PET Study. Ann. Neurol..

[19] Wang J., Huang S., Lan G., Lai Y.J., Wang Q.H., Chen Y., Xiao Z.S., Chen X., Bu X.L., Liu Y.H. (2025). Diagnostic accuracy of plasma p-tau217/Aβ42 for Alzheimer’s disease in clinical and community cohorts. Alzheimer’s Dement..

[20] Palmqvist S., Warmenhoven N., Anastasi F., Pilotto A., Janelidze S., Tideman P., Stomrud E., Mattsson-Carlgren N., Smith R., Ossenkoppele R. (2025). Plasma phospho-tau217 for Alzheimer’s disease diagnosis in primary and secondary care using a fully automated platform. Nat. Med..

[21] Viswan V., Shaffi N., Mahmud M., Subramanian K., Hajamohideen F. (2024). Explainable Artificial Intelligence in Alzheimer’s Disease Classification: A Systematic Review. Cogn. Comput..

[22] Ayton S., Diouf I. (2018). Bush AI for the Alzheimer’s disease Neuroimaging InitiativeEvidence that iron accelerates Alzheimer’s pathology: A CSF biomarker Studyjournal. Neurol. Neurosurg. Psychiatry.

[23] Konen F.F., Maier H.B., Neyazi A., Bleich S., Neumann K., Skripuletz T. (2023). Alzheimer’s disease biomarkers in cerebrospinal fluid are stable with the Elecsys immunoassay to most pre-analytical influencing factors except freezing at −80 °C. Neurol. Res. Pract..

[24] El-Sappagh S., Alonso J.M., Islam S.M.R., Sultan A.M., Kwak K.S. (2021). A multilayer multimodal detection and prediction model based on explainable artificial intelligence for Alzheimer’s disease. Sci. Rep..

[25] Yue L., Chen W.G., Liu S.C., Chen S.B., Xiao S.F. (2023). An explainable machine learning based prediction model for Alzheimer’s disease in China longitudinal aging study. Front. Aging Neurosci..

[26] Grammenos G., Vrahatis A.G., Vlamos P., Palejev D., Exarchos T., Alzheimer’s Disease Neuroimaging Initiative (2024). Predicting the Conversion from Mild Cognitive Impairment to Alzheimer’s Disease Using an Explainable AI Approach. Information.

[27] Jiao B., Ouyang Z., Xiao X., Zhang C., Xu T., Yang Q., Zhu Y., Liu Y., Liu X., Zhou Y. (2025). Development and validation of machine learning models with blood-based digital biomarkers for Alzheimer’s disease diagnosis: A multicohort diagnostic study. eClinicalMedicine.

[28] Christodoulou R., Vamvouras G., Lorentzen L., Vassiliou E. (2025). Erythrocyte Load in Cerebrospinal Fluid Linked with Hippocampal Atrophy in Alzheimer’s Disease. J. Clin. Med..

[29] Akiba T., Sano S., Yanase T., Ohta T., Koyama M. Optuna: A Next-generation Hyperparameter Optimization Framework. Proceedings of the 25th ACM SIGKDD International Conference on Knowledge Discovery & Data Mining.

[30] Cannistraro R.J., Badi M., Eidelman B.H., Dickson D.W., Middlebrooks E.H., Meschia J.F. (2019). CNS small vessel disease: A clinical review. Neurology.

[31] Katsi V., Marketou M., Maragkoudakis S., Didagelos M., Charalambous G., Parthenakis F., Tsioufis C., Tousoulis D. (2020). Blood–brain barrier dysfunction: The undervalued frontier of hypertension. J. Hum. Hypertens..

[32] Van Den Kerkhof M., De Jong J.J.A., Voorter P.H.M., Postma A.A., Kroon A.A., Van Oostenbrugge R.J., Jansen J.F., Backes W.H. (2024). Blood-Brain Barrier Integrity Decreases with Higher Blood Pressure: A 7T DCE-MRI Study. Hypertension.

[33] Bejanin A., Schonhaut D.R., La Joie R., Kramer J.H., Baker S.L., Sosa N., Ayakta N., Cantwell A., Janabi M., Lauriola M. (2017). Tau pathology and neurodegeneration contribute to cognitive impairment in Alzheimer’s disease. Brain.

[34] Ossenkoppele R., Schonhaut D.R., Schöll M., Lockhart S.N., Ayakta N., Baker S.L., O’Neil J.P., Janabi M., Lazaris A., Cantwell A. (2016). Tau PET patterns mirror clinical and neuroanatomical variability in Alzheimer’s disease. Brain.

[35] Rajpoot J., Crooks E.J., Irizarry B.A., Amundson A., Van Nostrand W.E., Smith S.O. (2022). Insights into Cerebral Amyloid Angiopathy Type 1 and Type 2 from Comparisons of the Fibrillar Assembly and Stability of the Aβ40-Iowa and Aβ40-Dutch Peptides. Biochemistry.

[36] Wang D., Chen F., Han Z., Yin Z., Ge X., Lei P. (2021). Relationship Between Amyloid-β Deposition and Blood–Brain Barrier Dysfunction in Alzheimer’s Disease. Front. Cell. Neurosci..

[37] Veszelka S., Tóth A.E., Walter F.R., Datki Z., Mózes E., Fülöp L., Bozsó Z., Hellinger E., Vastag M., Orsolits B. (2013). Docosahexaenoic Acid Reduces Amyloid-β Induced Toxicity in Cells of the Neurovascular Unit. J. Alzheimer’s Dis..

[38] Stokum J.A., Cannarsa G.J., Wessell A.P., Shea P., Wenger N., Simard J.M. (2021). When the Blood Hits Your Brain: The Neurotoxicity of Extravasated Blood. Int. J. Mol. Sci..

[39] Maheshwari S. (2023). Ferroptosis Signaling Pathways: Alzheimer’s Disease. Horm. Metab. Res..

[40] Hussain B., Fang C., Chang J. (2021). Blood–Brain Barrier Breakdown: An Emerging Biomarker of Cognitive Impairment in Normal Aging and Dementia. Front. Neurosci..

[41] Smith R., Strandberg O., Mattsson-Carlgren N., Leuzy A., Palmqvist S., Pontecorvo M.J., Devous Sr M.D., Ossenkoppele R., Hansson O. (2020). The accumulation rate of tau aggregates is higher in females and younger amyloid-positive subjects. Brain.

[42] Means J.C., Lopez A.A., Koulen P. (2021). Estrogen Protects Optic Nerve Head Astrocytes Against Oxidative Stress by Preventing Caspase-3 Activation, Tau Dephosphorylation at Ser422 and the Formation of Tau Protein Aggregates. Cell. Mol. Neurobiol..

[43] Huang Z., Wong L.W., Su Y., Huang X., Wang N., Chen H., Yi C. (2020). Blood-brain barrier integrity in the pathogenesis of Alzheimer’s disease. Front. Neuroendocrinol..

[44] Hsu T.M., Kanoski S.E. (2014). Blood-brain barrier disruption: Mechanistic links between Western diet consumption and dementia. Front. Aging Neurosci..

[45] Christodoulou R.C., Vamvouras G., Petrou V., Papageorgiou P.S., Pitsillos R., Rivera L., Vassiliou E., Papageorgiou S.G., Solomou E.E., Alzheimer’s Disease Neuroimaging Initiative (2025). Cerebrospinal Fluid Erythrocyte Burden Amplifies the Impact of PTAU on Entorhinal Degeneration in Alzheimer’s Disease. Biomolecules.

[46] Oomens J.E., van Gils V., Vos S.J.B., Freeze W.M., Maserejian N.N., Curiale G., Gillis C., Boada M., van der Flier W.M., Hort J. (2025). Cerebral Microbleeds and Amyloid Pathology Estimates from the Amyloid Biomarker Study. JAMA Netw. Open.

[47] Rauchmann B.S., Ghaseminejad F., Mekala S., Perneczky R., Alzheimer’s Disease Neuroimaging Initiative (2020). Cerebral Microhemorrhage at MRI in Mild Cognitive Impairment and Early Alzheimer Disease: Association with Tau and Amyloid β at PET Imaging. Radiology.

[48] Romero J.R., Demissie S., Beiser A., Himali J.J., DeCarli C., Levy D., Seshadri S. (2020). Relation of plasma β-amyloid, clusterin, and tau with cerebral microbleeds: Framingham Heart Study. Ann. Clin. Transl. Neurol..

[49] McCarter S.J., Lesnick T.G., Lowe V.J., Rabinstein A.A., Przybelski S.A., Algeciras-Schimnich A., Ramanan V.K., Jack C.R., Petersen R.C., Knopman D.S. (2022). Association Between Plasma Biomarkers of Amyloid, Tau, and Neurodegeneration with Cerebral Microbleeds. J. Alzheimer’s Dis..

[50] van Bergen J.M., Li X., Hua J., Schreiner S.J., Steininger S.C., Quevenco F.C., Wyss M., Gietl A.F., Treyer V., Leh S.E. (2016). Colocalization of cerebral iron with Amyloid beta in Mild Cognitive Impairment. Sci. Rep..

[51] Ayton S., Janelidze S., Kalinowski P., Palmqvist S., Belaidi A.A., Stomrud E., Roberts A., Roberts B., Hansson O., Bush A.I. (2023). CSF ferritin in the clinicopathological progression of Alzheimer’s disease and associations with APOE and inflammation biomarkers. J. Neurol. Neurosurg. Psychiatry.

[52] Spotorno N., Acosta-Cabronero J., Stomrud E., Lampinen B., Strandberg O.T., van Westen D., Hansson O. (2020). Relationship between cortical iron and tau aggregation in Alzheimer’s disease. Brain.

[53] FDA FDA Clears First Blood Test Used in Diagnosing Alzheimer’s Disease. https://www.fda.gov/news-events/press-announcements/fda-clears-first-blood-test-used-diagnosing-alzheimers-disease.

[54] Cummings J., Apostolova L., Rabinovici G.D., Atri A., Aisen P., Greenberg S., Hendrix S., Selkoe D., Weiner M., Petersen R.C. (2023). Lecanemab: Appropriate Use Recommendations. J. Prev. Alzheimer’s Dis..

[55] Rabinovici G.D., Selkoe D.J., Schindler S.E., Aisen P., Apostolova L.G., Atri A., Greenberg S.M., Hendrix S.B., Petersen R.C., Weiner M. (2025). Donanemab: Appropriate use recommendations. J. Prev. Alzheimer’s Dis..

[56] Ayton S., Barton D., Brew B., Brodtmann A., Clarnette R., Desmond P., Devos D., Ellis K.A., Fazlollahi A., Fradette C. (2025). Deferiprone in Alzheimer Disease: A Randomized Clinical Trial. JAMA Neurol..

[57] Ezkurdia A., Ramírez M.J., Solas M. (2023). Metabolic Syndrome as a Risk Factor for Alzheimer’s Disease: A Focus on Insulin Resistance. Int. J. Mol. Sci..

